# Age-Related Modifications of Electroencephalogram Coherence in Mice Models of Alzheimer’s Disease and Amyotrophic Lateral Sclerosis

**DOI:** 10.3390/biomedicines11041151

**Published:** 2023-04-11

**Authors:** Vasily Vorobyov, Alexander Deev, Kirill Chaprov, Aleksey A. Ustyugov, Ekaterina Lysikova

**Affiliations:** 1 School of Biosciences, Sir Martin Evans Building, Cardiff University, Museum Avenue, Cardiff CF10 3AX, UK; 2 Institute of Cell Biophysics, Russian Academy of Sciences, 142290 Pushchino, Russia; 3 Institute of Theoretical and Experimental Biophysics, Russian Academy of Sciences, 142290 Pushchino, Russia; 4 Institute of Physiologically Active Compounds, Russian Academy of Sciences, 142432 Chernogolovka, Russia; 5 Center of Pre-Clinical and Clinical Studies, Belgorod State National Research University, 308015 Belgorod, Russia

**Keywords:** aging, 5xFAD mice, FUS mice, electroencephalogram, coherence, cortex, hippocampus, putamen, ventral tegmental area, substantia nigra

## Abstract

Evident similarities in pathological features in aging and Alzheimer’s disease (AD) raise the question of a role for natural age-related adaptive mechanisms in the prevention/elimination of disturbances in interrelations between different brain areas. In our previous electroencephalogram (EEG) studies on 5xFAD- and FUS-transgenic mice, as models of AD and amyotrophic lateral sclerosis (ALS), this suggestion was indirectly confirmed. In the current study, age-related changes in direct EEG synchrony/coherence between the brain structures were evaluated. Methods: In 5xFAD mice of 6-, 9-, 12-, and 18-month ages and their wild-type (WT_5xFAD_) littermates, we analyzed baseline EEG coherence between the cortex, hippocampus/putamen, ventral tegmental area, and substantia nigra. Additionally, EEG coherence between the cortex and putamen was analyzed in 2- and 5-month-old FUS mice. Results: In the 5xFAD mice, suppressed levels of inter-structural coherence vs. those in WT_5xFAD_ littermates were observed at ages of 6, 9, and 12 months. In 18-month-old 5xFAD mice, only the hippocampus ventral tegmental area coherence was significantly reduced. In 2-month-old FUS vs. WT_FUS_ mice, the cortex–putamen coherence suppression, dominated in the right hemisphere, was observed. In 5-month-old mice, EEG coherence was maximal in both groups. Conclusion: Neurodegenerative pathologies are accompanied by the significant attenuation of intracerebral EEG coherence. Our data are supportive for the involvement of age-related adaptive mechanisms in intracerebral disturbances produced by neurodegeneration.

## 1. Introduction

Similar abnormalities observed in Alzheimer’s disease (AD) and aging [1] are suggested to be associated with disturbances in the functioning of neuronal networks in different brain structures or between them [2,3,4,5,6]. Some adaptive mechanisms preventing the functional decline during aging [7] are assumed to be involved in AD-associated processes [8]. Network alterations, in turn, are accompanied by evident changes in oscillatory features generated by the affected circuits [9,10]. Thus, electroencephalogram (EEG) recordings from various brain structures at different ages are expected to be a useful tool for the analysis of a role for adaptive mechanisms in the development of cerebral decline during the combined influence of AD and aging. Indeed, in previous studies on six- and twelve-month-old 5xFAD mice (an AD model) analyzing the frequency spectra of EEGs simultaneously recorded from cortical and subcortical areas, we assumed that the results might be explained by an involvement of adaptive/compensatory mechanisms in age-related AD pathology [11,12]. In two- and five-month-old FUS mice (a model of amyotrophic lateral sclerosis, ALS), the obtained data were in line with this suggestion [13]. However, relative changes in the frequency spectra of EEG recorded from different cerebral structures are indirect characteristics of their interrelations, whereas a coherence/synchrony of appearance of instantaneous values for EEGs [14] has been shown to be a direct and effective measure of “functional connectivity” in normal and diseased brains [15,16,17,18]. In particular, initial dysfunctions in cortical circuits have been shown to possibly initiate the ALS onset and drive its progression [19], whereas phasic changes in the functioning of neuronal circuits may characterize their ability to adapt as ALS develops [20]. Finally, the interaction of circuits with different connectivity and synchronized activities has been suggested to be involved in neurodegenerative and adaptive/compensatory changes in ALS [21,22]. 

To analyze an involvement of adaptive/compensatory processes in interrelations between AD and aging [7,8], we measured the levels of baseline EEG coherence between the cortex (MC), hippocampus (HPC)/striatum (putamen, Pt), ventral tegmental area (VTA), and substantia nigra (SN) in six-, eight-, twelve-, and eighteen-month-old 5xFAD mice versus their wild-type (WT_5xFAD_) littermates. In this study, coherence between EEGs from cortical and subcortical areas and from those containing dopamine neurons (VTA and SN) was measured given the evidence about the losing of these neurons in both AD groups [23], in particularly, in 5xFAD mice [11], and in WT_5xFAD_ (C57BL/6J) mice [24]. Additionally, we measured the levels of baseline EEG coherence between MC and Pt in two- and five-month-old FUS mice versus WT_FUS_ littermates with symmetrically implanted electrodes into these brain areas to control possible interhemispheric differences observed in normal and ALS-affected brains [25]. EEG coherence between MC and Pt is analyzed in this study because of an evolvement of basal ganglia–cortical functional connectivity at both normal development and pathological disturbances associated with basal ganglia dysfunctions [26].

## 2. Materials and Methods

In the “AD” groups, we used male transgenic mice with five familial AD mutations maintained on a C57BL/6J genetic background (5xFAD mice) at ages of 6, 9, 12, and 18 months (*n* = 11, 9, 9, and 7, respectively) and non-transgenic wild-type (WT_5xFAD_) littermates (*n* = 14, 8, 7, and 7, respectively). In the “ALS” groups, male transgenic mice with truncated human FUS lacking a nuclear localization signal maintained on the CD-1 genetic background (FUS mice, ΔFUS(1-359)) at ages of 2 and 5 months (*n* = 8 and 6, respectively) and WT_FUS_ littermates (*n* = 6 and 7, respectively) were used in this study. All mice were originally obtained from the Center for Collective Use of the Institute of Physiologically Active Compounds RAS (Chernogolovka, Russian Federation). Mice were housed in a standard environment (12 h light/dark cycle, 22–25 °C RT, 50–55% relative humidity) with food and water ad libitum. The procedures were carried out in compliance with the principles enunciated in the Directive 2010/63/EU on the protection of animals used for scientific purposes, and approved by the local institute’s ethics review committee. All efforts were made to minimize the number of animals and their suffering.

### 2.1. Implantation of Electrodes

Each mouse was narcotized by subcutaneous injection of the mixture of tiletamine/zolazepam (25 mg/kg, Zoletil^®^, Virbac, France) and xylazine (2.5 mg/kg, Rometar^®^, Bioveta, Czech Republic). In the 5xFAD mice, EEG recording electrodes were positioned in the left motor cortex and dorsal hippocampus (MC and HPC; AP: +1.1; ML: 1.5; DV: −0.75 and AP: −2.8; ML: −2.7; DV: −1.7 mm, respectively) or putamen (Pt; AP: +1.1; ML: 1.5; DV: −2.75 mm, in “12-month” group), in the left ventral tegmental area (VTA; AP: −3.1; ML: −0.4; DV: −4.5), and in the right substantia nigra (SN; AP: −3.2, ML: +1.3, DV: −4.3) accordantly with the mouse brain atlas [27], where AP, ML, and DV are the distances from the coronal suture, midline suture, and skull surface, respectively). Within the brain areas analyzed in this study, the opposite hemisphere for SN was chosen to exclude possible mutual damage during electrode implantations in the same hemisphere because of SN proximity to VTA. Furthermore, it is well known that the contralateral SN is the dominant source of DA in the opposite hemisphere.

Two varnish-insulated nichrom wires (100 µm in diameter) glued together (3M VetbondTM Tissue Adhesive, St. Paul, MN, USA) with both tips free from insulation for 100 µm were used during the preparation of electrodes for EEG recordings from all brain structures. One of wires in the electrode for EEG recordings from MC and Pt was shortened by 1 mm to simultaneously reach both brain areas. Thus, the prepared electrodes were sufficiently inflexible and had a more beneficial surface–volume ratio than a single-wire electrode of 200 µm diameter. The reference and ground electrodes (stainless steel wire, 0.4 mm in diameter) were implanted over the caudal part of the brain (AP: −5.3; ML: ±1.8; DV: −0.5). A computerized 3D stereotaxic StereoDrive (Neurostar, Tübingen, Germany) was used for the precise positioning of all electrodes. The latter were fixed to the skull with dental cement and soldered to a dual row socket connector (Sullins Connector Solutions, San Marcos, CA, USA). Each of the nichrom wires was soldered to one of the connector’s pins. After electrode implantation, mice were housed individually for the recovery, followed by the experimental sessions and postmortem verification of the electrode tip location (see the examples and histological details in [11,12]). 

### 2.2. EEG Recording

Three–four days after implantation of the electrodes, each mouse was allowed to adapt for several days (one hour per day) to the experimental environment, including an internal cage (Perspex, 15 cm × 17 cm × 20 cm) taken place in an electrically shielded chamber and a cable (five 36- gauge wires, Plexon Inc, Dallas, TX, USA) plugged in a digital Neuro-MEP amplifier (Neurosoft Ltd., Ivanovo, Russian Federation). Baseline EEG was recorded on day 8 for 30 min, starting after 20–30-min adaptation of the animal to the experimental cage. The experiments were performed during a daylight period (9:00 a.m.–6:00 p.m.), keeping the illumination at a relatively stable level by combination with an artificial light source if it was necessary.

### 2.3. EEG Coherence Computation

**Figure 1:**
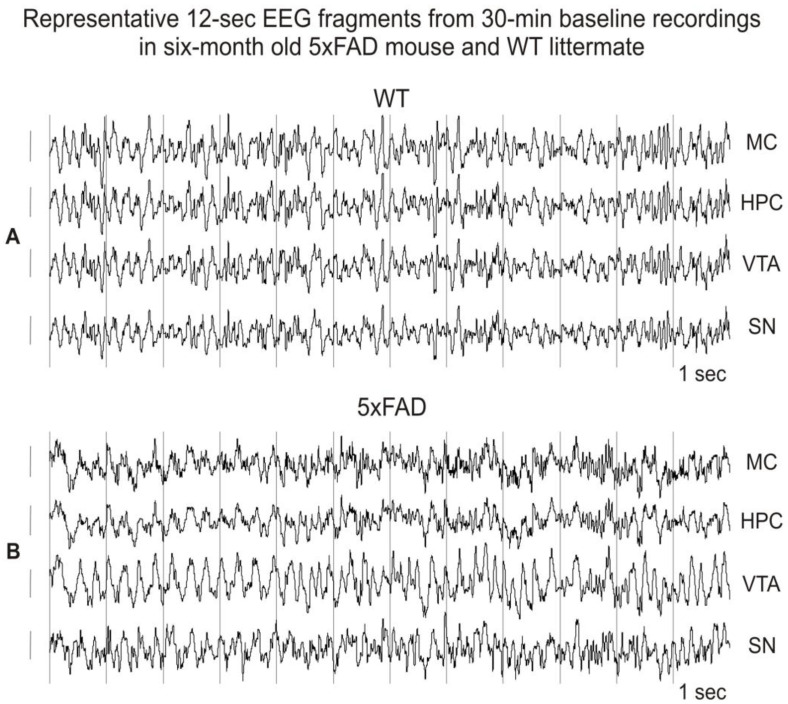
Typical patterns in 12 s fragments of baseline EEG in wakeful and behaviorally active six-month-old WT_5xFAD_ mouse and 5xFAD littermate (**A** and **B,** respectively) in the motor cortex (MC), hippocampus (HPC), ventral tegmental area (VTA), and substantia nigra (SN). On **A** and **B**, time calibration is 1 sec, amplitude calibration is 100 µV.

EEG signals were amplified, filtered (0.1–35 Hz), and sampled (1 kHz) online using the amplifier and kept in the memory of an operational computer for further analysis. The program allowed for both the automatic and manual rejection of EEG fragments containing artifacts and epileptic spikes. In the raw EEGs, evident differences in cerebral synchronization were observed between 5xFAD and WT_5xFAD_ mice (Figure 1). 

**Figure 2:**
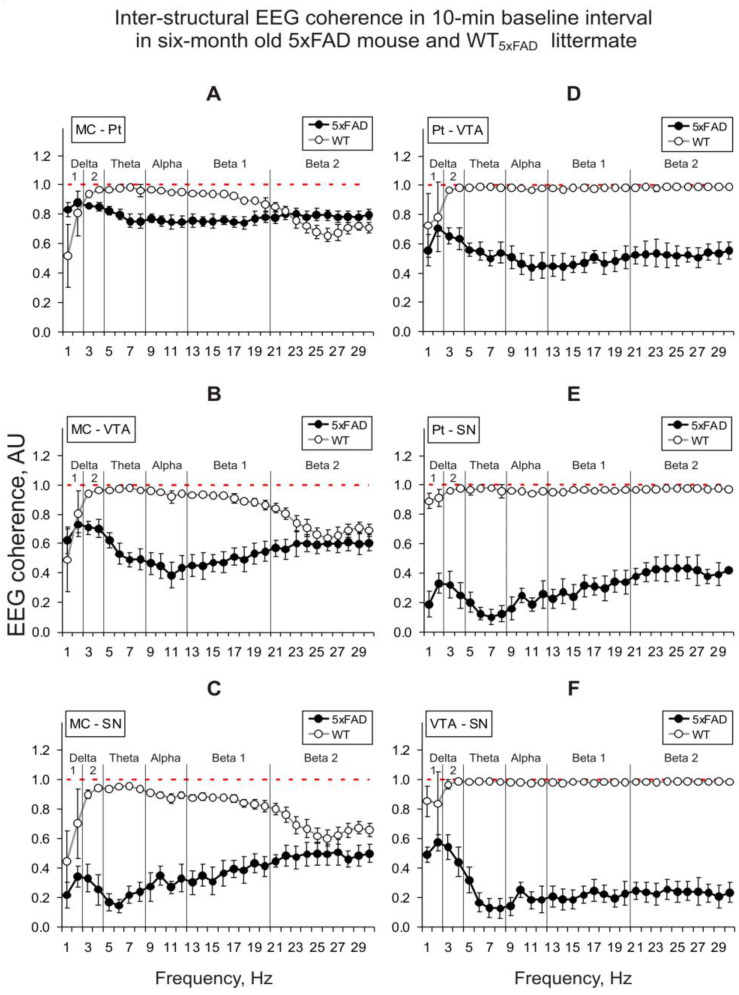
Inter-structural coherence distributions (**A**–**F**) in six-month-old 5xFAD mouse and WT_5xFAD_ littermate (filled and open circles, respectively) in 10 min baseline EEGs from the motor cortex (MC), hippocampus (HPC), ventral tegmental area (VTA), and substantia nigra (SN), in different frequency bands denoted on horizontal axis (abscissa). Inter-structural coherence is denoted on the plates as MC-HPC (**A**); MC-VTA (**B**); MC-SN (**C**); HPC-VTA (**D**); HPC-SN (**E**), and VTA-SN (**F**). Ordinate is the average value of EEG coherence in each of 1 Hz bands. Five vertical lines separate “classical” EEG frequency bands (from left to right: delta 1, delta 2, theta, alpha, beta 1, and beta 2, respectively). Red dashed lines show maximal coherence value.

EEG data were processed using custom prepared software (see Attachment A1). Spectral coherence was estimated by averaging over 12 s epochs of baseline EEGs derived from a 1–30 Hz range, initially in a 1 Hz wide band (Figure 2). 

Afterwards, EEG coherence values were evaluated in the range of 1–30 Hz with the averaging of data in “classical” EEG bands (in Hz): delta 1 (1–2), delta 2 (2–4), theta (4–8), alpha (8–12 Hz), beta 1 (12–20), and beta 2 (20–30). The values of coherence in each of the frequency bands were averaged in each of the three consecutive 10 min intervals (for further statistical analysis) and for 30 min in total (for illustrations).

### 2.4. Statistics

Differences between transgenic and non-transgenic mice in the EEG coherency averaged in each of three consecutive 10 min intervals in frames of both the whole frequency spectra and individual frequency bands were analyzed by two-way ANOVA for repeated measures with Bonferroni’s post hoc test for multiple comparisons (STATISTICA 10; StatSoft, Inc., Tulsa, OK, USA). All sets of data were preliminary tested on Gaussian distribution. There were 36 parameters for coherence: six “classical” frequency bands X six combinations of inter-structural coherence. Results were considered statistically significant at *p* < 0.05. All data are shown as mean ± SEM.

## 3. Results

During baseline EEG recordings, the behavior of mice was typically characterized by relatively intensive exploration of the experimental box, which was occasionally alternated by short-lasting calm down periods. Baseline EEGs and their frequency compositions have been described in detail in our previous studies [11,12,13].

### 3.1. EEG Coherence in 5xFAD Mice of Different Ages

**Figure 3:**
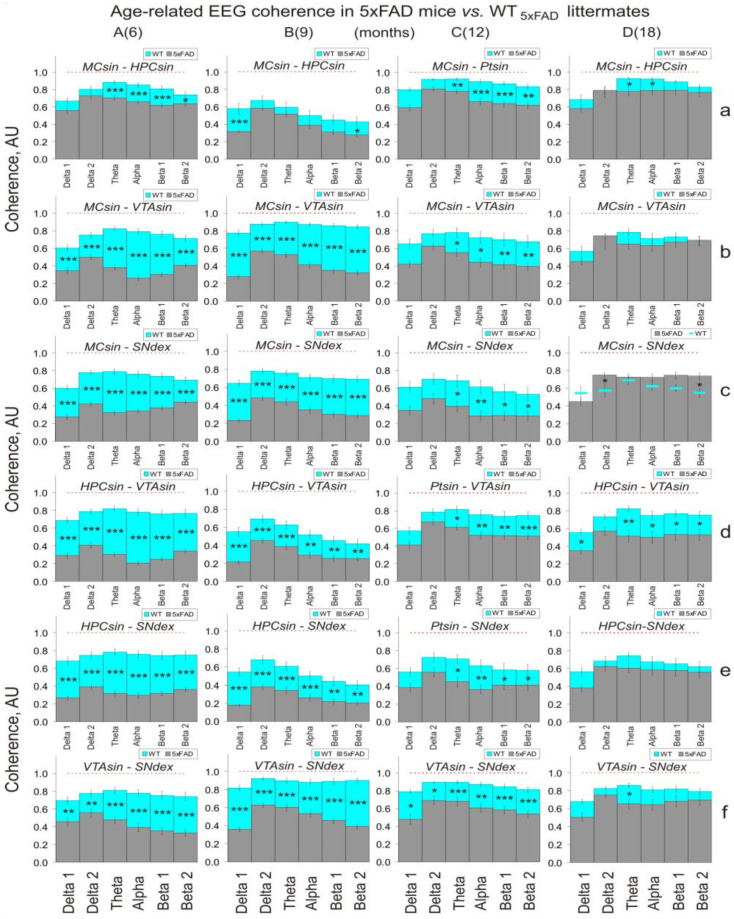
Age-related relations between coherence distributions in 5xFAD and WT_5xFAD_ mice (grey and light blue bars, respectively) for 30 min baseline EEG from the motor cortex (MC), hippocampus (HPC)/putamen (Pt), ventral tegmental area (VTA), and substantia nigra (SN), in different frequency bands. Upper- and lower-case letters are used to recognize any age (**A**–**D**) and the inter-structural relations (**a**–**f**) denoted on the plates in brackets and by italic fonts, respectively. Ordinate is the average value of EEG coherence in each of the “classical” bands denoted on the horizontal axes (abscissa). Red dashed lines show maximal coherence value. Abbreviations of “sin” and “dex” indicate “left” and “right” hemispheres, respectively. Star symbols denote significant two-way ANOVA differences in separate frequency bands between 5xFAD and WT5xFAD mice, where *, **, and *** correspond to *p* < 0.05, *p* < 0.01, and *p* < 0.001, respectively (see Figure A1, for details).

Differences in the levels of EEG coherence between 5xFAD-mice and their WT_5xFAD_ littermates were relatively stable in consecutive 10 min intervals that were readily visible in the spectral profiles of coherence averaged in the whole (30 min) baseline period that was expressed in very small values of SEMs (Figure 3). 

The 5xFAD mice showed significantly lower levels of EEG coherence vs. the WT_5xFAD_ littermates in most of the analyzed frequency bands (c.f., grey and light blue bars in Figure 3).

Evident age-related attenuation of the coherence differences between the groups was observed. The differences were significantly higher in younger mice (two-way ANOVA: (a) *6-month-old mice*: F_138_ = 17.9, 105, 107, 163, 96.4, and 46.3, for MC-HPC, MC-VTA, MC-SN; HPC-VTA, HPC-SN; and VTA-SN, respectively, *p* < 0.001, for all; (b) *9-month-old mice*: F_90_ = 7.8 and 233, 90.7, 29.2, 36.6, 115, *p* = 0.006, for MC-HPC and *p* < 0.001, for others, respectively; (c) *12-month-old mice*: F_84_ = 11.2, 11.1, 10.8, 11.5, 10.9, and 17.7, *p* = 0.001, 0.001, 0.002, 0.001, 0.001, and <0.001, respectively). The coherence differences practically disappeared in the *18-month-old mice*, with the exception of HPC-VTA and VTA-SN interrelations: F_72_ = 1.1, 0.3, 3.6, 13.4, 0.6, and 9.4, *p* = 0.299, 0.588, 0.063, <0.001, 0.440, and 0.003, respectively (Figure 3D,d; see Figure S2 for clarity). The elimination of intracerebral coherence differences between the 18-month-old 5xFAD and WT_5FAD_ mice was evidently associated with the age-related recovery of EEG synchrony in the transgenic mice. Indeed, at the age of 18 months, 5xFAD mice demonstrated a more significant rising of EEG coherence vs. that in the younger mice (c.f., grey bars in Figure 3D vs. Figure 3A–C; two-way ANOVA: (a) D vs. A: F_96_ = 13.1, 53.8, 127, 33.4, 52.1, and 23, for MC-HPC, MC-VTA, MC-SN; HPC-VTA, HPC-SN; and VTA-SN, respectively, *p* < 0.001 for all; (b) D vs. B: F_84_ = 110, 39, 97, 26.7, 69, and 17.6, respectively, *p* < 0.001, for all; (c) D vs. C: F_84_ = 6.24, 14.9, 46, 0.04, 11.8, and 2.5, *p* = 0.014, <0.001, <0.001, 0.846, 0.001, and 0.117, respectively). 

### 3.2. EEG Coherence in FUS Mice of Two- and Five-Month Ages

**Figure 4:**
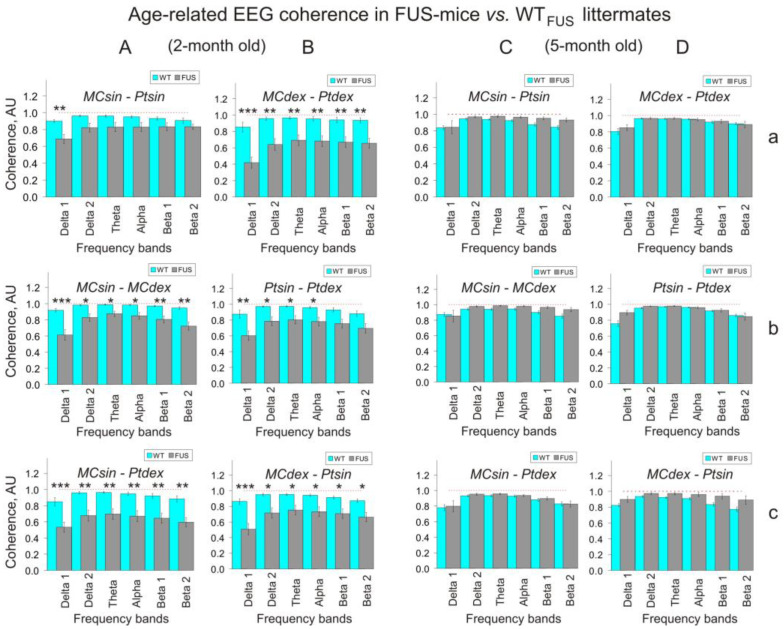
Age-dependent relations between coherence distributions in FUS and WT_FUS_ mice (grey and light blue bars, respectively) for 30 min baseline EEG from symmetrical motor cortex (MC) and putamen (Pt), in different frequency bands. Upper-case letters are used to recognize FUS mice (**A**,**C**) and WT littermates (**B**,**D**) at different ages (see in brackets) and the inter-structural relations (**a**–**c**) denoted on the plates by italic fonts. Ordinate is the average value of EEG coherence in each of the “classical” bands denoted on the horizontal axes (abscissa). Abbreviations of “sin” and “dex” indicate “left” and “right” hemispheres, respectively. Red dashed lines show maximal coherence value. Star symbols denote significant two-way ANOVA differences in separate frequency bands between FUS and WT_FUS_ mice, where *, **, and *** correspond to *p* < 0.05, *p* < 0.01, and *p* < 0.001, respectively (see Figure A2, for details).

The differences in the levels of EEG coherence between FUS mice and their WT_FUS_- littermates were relatively stable in consecutive 10 min intervals that were readily visible in the spectral profiles of coherence averaged in the whole (30 min) baseline period that was expressed in extremely small values of SEMs (Figure 4). 

During this period, EEG coherence between MC and Pt in two-month-old FUS mice was significantly less of that in WT_FUS_ littermates practically in all frequency bands regardless of the brain hemisphere (Figure 4A,B; two-way ANOVA: F_72_ = 7.3, 16.3, 21.8, 23.2, 12.7, and 15.1, for Mcsin-Ptsin, Mcsin-MCdex, Mcsin-Ptdex, Mcdex-Ptdex, Ptsin-Ptdex, and Mcdex-Ptsin, respectively; *p* = 0.008 and <0.001, for others). Interestingly, in the FUS mice, MC-Pt coherence in the left hemisphere was significantly higher to that on the right side (c.f., grey bars in Figure 2A,B,a; two-way ANOVA: F_1,84_ = 7.26, *p* = 0.008). The EEG coherence suppression, observed in younger FUS mice, completely disappeared in the older ones (see Figure 4C,D and Figure S2, for clarity). 

## 4. Discussion

In this study, the age-related suppression of coherence between EEGs from different brain structures in both 5xFAD and FUS mice was revealed (see Figure 3 and Figure 4, respectively). This phenomenon has been shown to be characteristic for AD [17,28,29,30]. However, we demonstrate that the EEG coherence suppression is age-dependent, which allows the development of a “functional connectivity” approach for the studying of a role for compensatory/adaptive mechanisms in the assessment and correction of age-related AD evolution. Aging and AD are well known to be characterized by a gradual cognitive deterioration associated with modifications in the brain structures and their functional interaction [31,32] that in turn is accompanied by disturbances in neuronal synchrony [33,34,35,36]. In the aging brain, the role of the so-called “repressor element silencing transcription factor” (REST), which is well known to initiate a specific “stress-response program”, has been highlighted to be preventive for cognitive decline and AD [8,37]. The REST protein, normally expressed at low levels in the neurons of young brains, has been shown to be profoundly elevated in aged brains [8]. In 5xFAD mice, this might be linked with the age-dependent accumulation of synaptosomal mitochondrial dysfunctions [38]. In line with this suggestion, the accumulation of mitochondrial DNA deletions in dopaminergic neurons has been shown to trigger neuroprotective compensatory mechanisms [39]. In our study, the genetically programmed AD is expected to be a powerful “stressor” for the REST’s production, noticeably exceeding that in the aging process alone. Age-dependent suppression of EEG coherence in 5xFAD mice (see Figure 3) seems to characterize the REST involvement in a recovery of interrelations between the affected brain structures up to those in control 18-month-old WT_5xFAD_ mice. Residual suppression of HPC-VTA coherence in 5xFAD mice at this age (Figure 3d,D) highlights enhanced susceptibility of interrelations between these structures to AD-associated pathological disturbances in the brain. Given the important modulatory role of VTA in the switching of HPC from inhibition to the enhancement of its information flow [40], the residual suppression of EEG coherence between VTA and HPC might be a part of age-related mechanisms of cognitive impairment in AD [41]. The suppressed EEG synchrony/coherence between VTA and SN are seemingly linked with the age-related loss of dopaminergic neurons in these areas, where a statistically significant effect has been shown to start from 9 months of age [41]. A similar phenomenon was observed in WT_5xFAD_ (C57BL/6) littermates [24]. The age-related expansion of microglia into the dopamine-producing areas is directed to increase dopamine neuron surveillance by compensating for the progressive decline in morphological complexity (senescence) of microglia [42,43]. Thus, an association of senescent cells, brain plasticity and impairments in cognition might be an attractive target for further studies of interrelations between mechanisms of aging and AD [44].

In younger (two-month-old) FUS mice vs. control WT_FUS_ (CD-1) littermates, evident EEG coherence suppression was observed in all cortex–putamen combinations (see Figure 3A,B). In older transgenic mice, the coherence profiles were practically identical to those in non-transgenic ones (see Figure 3C,D). This is in line with the suggestion that a combination of circuits with different connectivity is characteristic for an involvement of neurodegenerative and adaptive mechanisms in ALS development [21]. Interestingly, in two-month-old FUS mice, EEG coherence between MC and Pt in the left hemisphere was noticeably higher than that on the right side (c.f., Figure 4a,A vs. Figure 4B), despite the lacking differences in interhemispheric asymmetry separately in the cortices and putamen (c.f., Figure 4b,A,B, respectively). This early characteristic of ALS might be explained by the impairment of coupled structural and functional connectivity during the development of the disease [45], thus highlighting the role of cortico–striatal interrelations in age-related ALS mechanism(s). On the other hand, this allows the development of a special asymmetry-based approach for the differentiation of ALS from other neurodegenerative pathologies. The intimate age-related mechanisms of ALS pathogenesis seem to be linked with a fine balance in the expression of inflammation-associated factors that in turn affects the developmental profile of disease and survival [46,47]. EEG coherence recovery in five-month-old FUS mice to control values (Figure 4C,D) is in line with protective microglia functions from ALS-related degeneration that is observed, in particular, in TDP-43 mice, one of the ALS models [48].

In spite of evident total suppression of EEG coherence in younger 5xFAD and FUS mice, some peculiarities revealed in this study might be preliminarily analyzed. Shortly, different levels of coherence between EEGs from the brain areas in WT_5xFAD-_ and WT_FUS_- mice (c.f., light blue bars in Figure 3 and Figure 4) appear to be associated with different temporal patterns of involvement of pro- and anti-inflammatory factors in mechanisms of adaptation in mice of different phenotypes (C57BL/6J and CD-1, respectively). Secondly, suppressed EEG coherence between MC and Pt in the right vs. left hemispheres in two-month-old FUS mice (c.f., grey bars in Figure 4a,B vs. Figure 4A, respectively) highlights the significance of intra-hemispheric functional interrelations in addition to inter-hemispheric asymmetry shown in other ALS studies [25]. Interestingly, no interhemispheric asymmetry was separately observed for MC and Pt in our study (c.f., grey bars in Figure 4b,A,B). Finally, evidently less EEG coherence between HPC and other brain areas in nine-month-old WT_5xFAD_ mice (c.f., Figure 3a,d,e,B vs. Figure 3A,C,D) highlights an age dependence of a role of HPC in the brain functioning, specifically, of cognitive mechanisms in mice [24]. 

## 5. Conclusions

Our data highlight a crucial role of age-related adaptive mechanisms in the modification/elimination of intracerebral disturbances associated with neurodegenerative pathologies (AD and ALS, in particular). We demonstrate that intra-cerebral coherence measurements allow the revealing of the pathologies, their differentiation, and progression. This approach is apparently useful and effective for the understanding of the inter-structural mechanisms involved in the development of neurodegenerative processes of different etiologies. In this respect, further detailed analysis of a role for frequency-specific EEG oscillations in the functional coupling of different brain areas has a great potential for the understanding of the interconnected mechanisms of adaptation and pathology. 

## Figures and Tables

**Figure A1:**
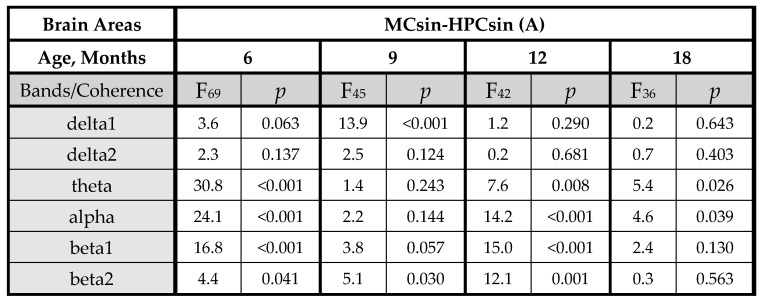

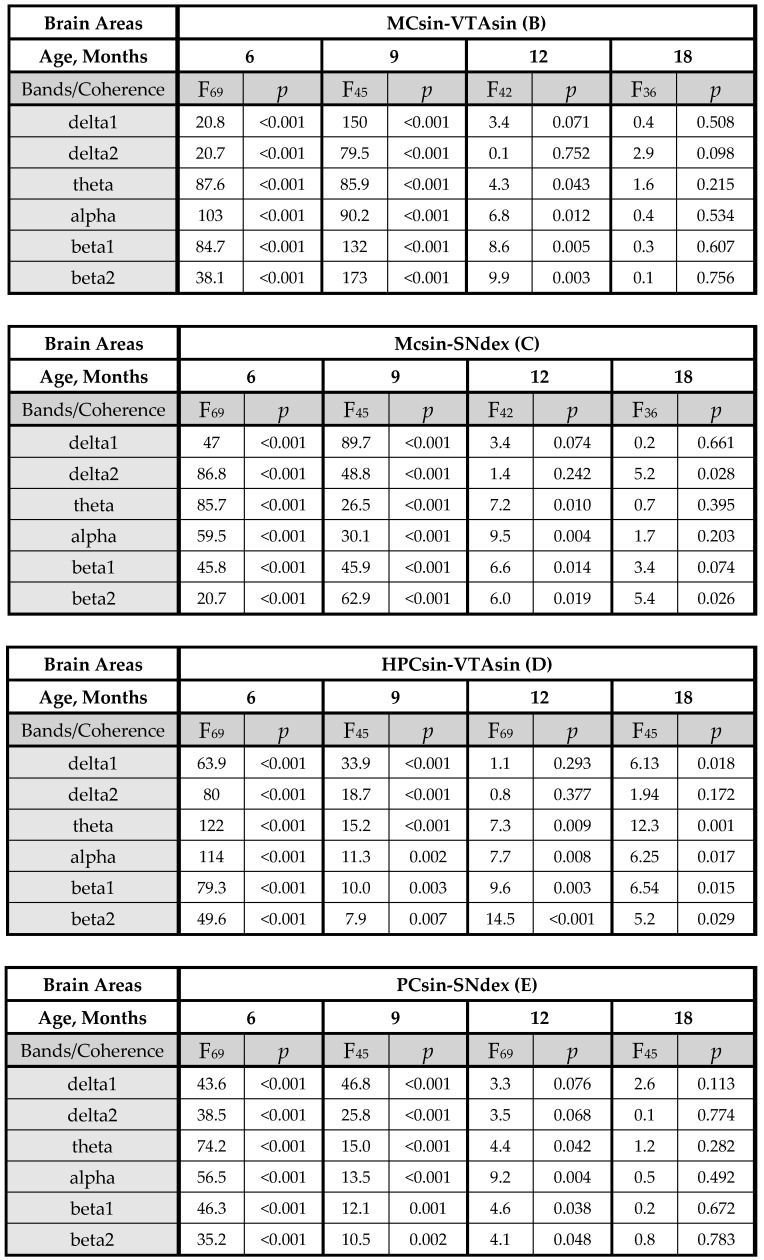

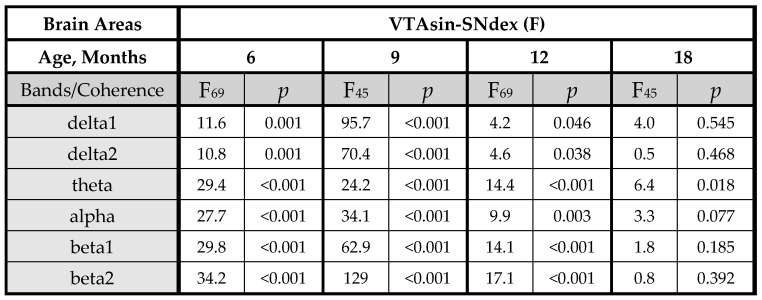
Two-way ANOVA analysis of EEG coherence in separate frequency bands between various brain areas (**A**–**F**) in 5xFAD vs. WT_5xFAD_ mice of different ages (6, 9, 12, 18 months).

**Figure A2:**
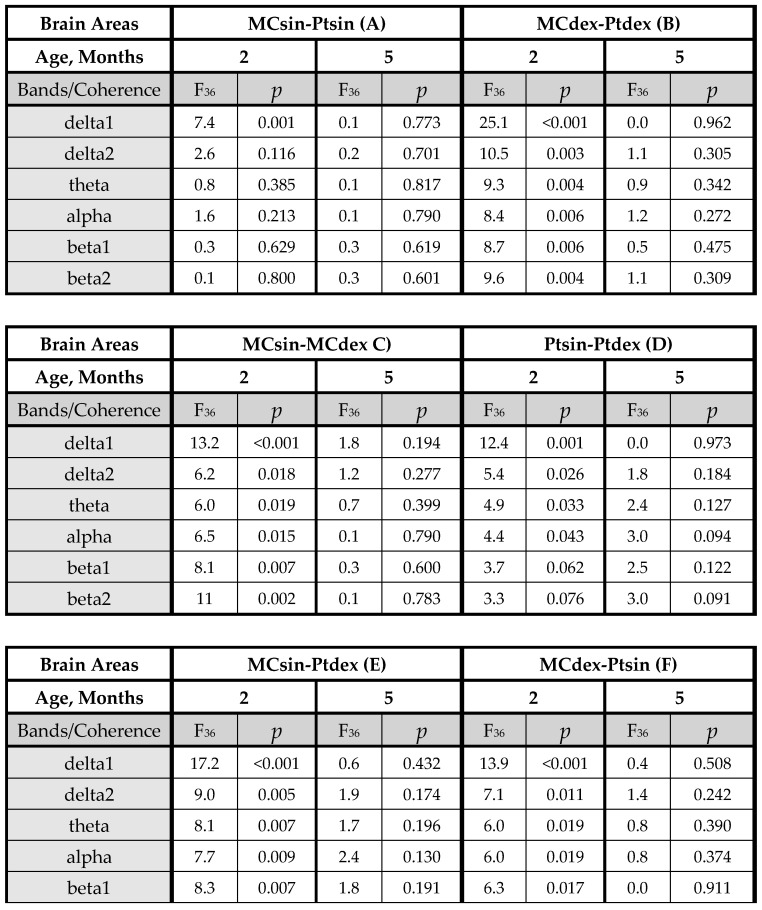

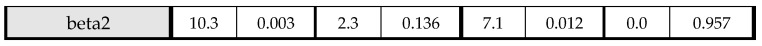
Two-way ANOVA analysis of EEG coherence in separate frequency bands between various brain areas (**A**–**F**) in FUS vs. WT_FUS_ mice of different ages (2 and 5 months).

## Data Availability

Data are contained within the current article and its Supplementary Material.

## Supplementary Materials

The following supporting information can be downloaded at https://www.mdpi.com/article/10.3390/biomedicines11041151/s1, Figure S1—Short description of custom prepared soft for calculation of EEG coherence function; Figure S2—Age-related differences between EEG coherence distributions in 5xFAD and WT_5xFAD_ mice estimated by two-way ANOVA; Figure S3—Age-related differences between EEG coherence distributions in FUS and WT_FUS_ mice estimated by two-way ANOVA. Refs. [49,50,51] are cited in the supplementary materials.

## References

[1] Peng S., Zeng L., Haure-Mirande J.V., Wang M., Huffman D.M., Haroutunian V., Ehrlich M.E., Zhang B., Tu Z. (2021). Transcriptomic Changes Highly Similar to Alzheimer’s Disease Are Observed in a Subpopulation of Individuals during Normal Brain Aging. Front. Aging Neurosci..

[2] Wang P., Zhou B., Yao H., Zhan Y., Zhang Z., Cui Y., Xu K., Ma J., Wang L., An N. (2015). Aberrant intra- and inter-network connectivity architectures in Alzheimer’s disease and mild cognitive impairment. Sci. Rep..

[3] Canter R.G., Penney J., Tsai L.H. (2016). The road to restoring neural circuits for the treatment of Alzheimer’s disease. Nature.

[4] Wu Z., Gao Y., Potter T., Benoit J., Shen J., Schulz P.E., Zhang Y., The Alzheimer’s Disease Neuroimaging Initiative (2021). Interactions between Aging and Alzheimer’s Disease on Structural Brain Networks. Front. Aging Neurosci..

[5] Babiloni C., Blinowska K., Bonanni L., Cichocki A., De Haan W., Del Percio C., Dubois B., Escudero J., Fernández A., Frisoni G. (2020). What electrophysiology tells us about Alzheimer’s disease: A window into the synchronization and connectivity of brain neurons. Neurobiol. Aging.

[6] Watanabe H., Bagarinao E., Maesawa S., Hara K., Kawabata K., Ogura A., Ohdake R., Shima S., Mizutani Y., Ueda A. (2021). Characteristics of Neural Network Changes in Normal Aging and Early Dementia. Front. Aging Neurosci..

[7] Aron L., Zullo J., Yankner B.A. (2021). The adaptive aging brain. Curr. Opin. Neurobiol..

[8] Tsai L.H., Madabhushi R. (2014). A protective factor for the ageing brain. Nature.

[9] Nimmrich V., Draguhn A., Axmacher N. (2015). Neuronal Network Oscillations in Neurodegenerative Diseases. Neuromol. Med..

[10] Palop J.J., Mucke L. (2016). Network abnormalities and interneuron dysfunction in Alzheimer disease. Nat. Rev. Neurosci..

[11] Vorobyov V., Bakharev B., Medvinskaya N., Nesterova I., Samokhin A., Deev A., Tatarnikova O., Ustyugov A.A., Sengpiel F., Bobkova N. (2019). Loss of Midbrain Dopamine Neurons and Altered Apomorphine EEG Effects in the 5xFAD Mouse Model of Alzheimer’s Disease. J. Alzheimer’s Dis..

[12] Vorobyov V., Deev A., Oganesyan Z., Sengpiel F., Ustyugov A.A. (2022). Baseline Electroencephalogram and Its Evolution after Activation of Dopaminergic System by Apomorphine in Middle-Aged 5XFAD Transgenic Mice, a Model of Alzheimer’s Disease. Dynamics.

[13] Vorobyov V., Deev A., Sengpiel F., Nebogatikov V., Ustyugov A.A. (2021). Cortical and Striatal Electroencephalograms and Apomorphine Effects in the FUS Mouse Model of Amyotrophic Lateral Sclerosis. J. Alzheimer’s Dis..

[14] Nunez P.L., Srinivasan R. (2006). Electric Fields of the Brain: The Neurophysics of EEG.

[15] Cook I.A., Leuchter A.F. (1996). Synaptic dysfunction in Alzheimer’s disease: Clinical assessment using quantitative EEG. Behav. Brain Res..

[16] Sankari Z., Adeli H., Adeli A. (2011). Intrahemispheric, interhemispheric, and distal EEG coherence in Alzheimer’s disease. Clin. Neurophysiol..

[17] Wang R., Wang J., Yu H., Wei X., Yang C., Deng B. (2015). Power spectral density and coherence analysis of Alzheimer’s EEG. Cogn. Neurodyn..

[18] Abazid M., Houmani N., Boudy J., Dorizzi B., Mariani J., Kinugawa K. (2021). Comparative Study of Functional Connectivity Measures for Brain Network Analysis in the Context of AD Detection with EEG. Entropy.

[19] Brunet A., Stuart-Lopez G., Burg T., Scekic-Zahirovic J., Rouaux C. (2020). Cortical Circuit Dysfunction as a Potential Driver of Amyotrophic Lateral Sclerosis. Front. Neurosci..

[20] Kim J., Hughes E.G., Shetty A.S., Arlotta P., Goff L.A., Bergles D.E., Brown S.P. (2017). Changes in the Excitability of Neocortical Neurons in a Mouse Model of Amyotrophic Lateral Sclerosis Are Not Specific to Corticospinal Neurons and Are Modulated by Advancing Disease. J. Neurosci..

[21] Abidi M., de Marco G., Couillandre A., Feron M., Mseddi E., Termoz N., Querin G., Pradat P.F., Bede P. (2020). Adaptive functional reorganization in amyotrophic lateral sclerosis: Coexisting degenerative and compensatory changes. Eur. J. Neurol..

[22] Bede P., Bogdahn U., Lope J., Chang K.M., Xirou S., Christidi F. (2021). Degenerative and regenerative processes in amyotrophic lateral sclerosis: Motor reserve, adaptation and putative compensatory changes. Neural Regen. Res..

[23] Nobili A., Latagliata E.C., Viscomi M.T., Cavallucci V., Cutuli D., Giacovazzo G., Krashia P., Rizzo F.R., Marino R., Federici M. (2017). Dopamine neuronal loss contributes to memory and reward dysfunction in a model of Alzheimer’s disease. Nat. Commun..

[24] Noda S., Sato S., Fukuda T., Tada N., Hattori N. (2020). Aging-related motor function and dopaminergic neuronal loss in C57BL/6 mice. Mol. Brain.

[25] Karandreas N., Papadopoulou M., Kokotis P., Papapostolou A., Tsivgoulis G., Zambelis T. (2007). Impaired interhemispheric inhibition in amyotrophic lateral sclerosis. Amyotroph. Lateral Scler..

[26] Greene D.J., Laumann T.O., Dubis J.W., Ihnen S.K., Neta M., Power J.D., Pruett J.R., Black K.J., Schlaggar B.L. (2014). Developmental changes in the organization of functional connections between the basal ganglia and cerebral cortex. J. Neurosci..

[27] Franklin K.B.J., Paxinos G. (2007). The Mouse Brain in Stereotaxic Coordinates.

[28] Hogan M.J., Swanwick G.R., Kaiser J., Rowan M., Lawlor B. (2003). Memory-related EEG power and coherence reductions in mild Alzheimer’s disease. Int. J. Psychophysiol..

[29] Stevens A., Kircher T., Nickola M., Bartels M., Rosellen N., Wormstall H. (2001). Dynamic regulation of EEG power and coherence is lost early and globally in probable DAT. Eur. Arch. Psychiatry Clin. Neurosci..

[30] Ahnaou A., Moechars D., Raeymaekers L., Biermans R., Manyakov N.V., Bottelbergs A., Wintmolders C., Van Kolen K., Van De Casteele T., Kemp J.A. (2017). Emergence of early alterations in network oscillations and functional connectivity in a tau seeding mouse model of Alzheimer’s disease pathology. Sci. Rep..

[31] Valenzuela M.J., Breakspear M., Sachdev P. (2007). Complex mental activity and the aging brain: Molecular, cellular and cortical network mechanisms. Brain Res. Rev..

[32] De Strooper B., Karran E. (2016). The cellular phase of Alzheimer’s disease. Cell.

[33] Uhlhaas P., Singer W. (2006). Neural synchrony in brain disorders: Relevance for cognitive dysfunctions and pathophysiology. Neuron.

[34] Palop J.J., Mucke L. (2010). Amyloid-β-induced neuronal dysfunction in Alzheimer’s disease: From synapses toward neural networks. Nat. Neurosci..

[35] Womelsdorf T., Schoffelen J.M., Oostenveld R., Singer W., Desimone R., Engel A.K., Fries P. (2007). Modulation of neuronal interactions through neuronal synchronization. Science.

[36] Koenig T., Prichep L., Dierks T., Hubl D., Wahlund L.O., John E.R., Jelic V. (2005). Decreased EEG synchronization in Alzheimer’s disease and mild cognitive impairment. Neurobiol. Aging.

[37] Lu T., Aron L., Zullo J., Pan Y., Kim H., Chen Y., Yang T.H., Kim H.M., Drake D., Liu X.S. (2014). REST and stress resistance in ageing and Alzheimer’s disease. Nature.

[38] Wang L., Guo L., Lu L., Sun H., Shao M., Beck S.J., Li L., Ramachandran J., Du Y., Du H. (2016). Synaptosomal Mitochondrial Dysfunction in 5xFAD Mouse Model of Alzheimer’s Disease. PLoS ONE.

[39] Perier C., Bender A., García-Arumí E., Melià M.J., Bové J., Laub C., Klopstock T., Elstner M., Mounsey R.B., Teismann P. (2013). Accumulation of mitochondrial DNA deletions within dopaminergic neurons triggers neuroprotective mechanisms. Brain.

[40] Rosen Z.B., Cheung S., Siegelbaum S.A. (2015). Midbrain dopamine neurons bidirectionally regulate CA3-CA1 synaptic drive. Nat. Neurosci..

[41] Fertan E., Brown R.E. (2022). Age-related deficits in working memory in 5xFAD mice in the Hebb-Williams maze. Behav. Brain Res..

[42] Shaerzadeh F., Phan L., Miller D., Dacquel M., Hachmeister W., Hansen C., Bechtle A., Tu D., Martcheva M., Foster T.C. (2020). Microglia senescence occurs in both substantia nigra and ventral tegmental area. Glia.

[43] Nekrasov P.V., Vorobyov V.V. (2018). Dopaminergic mediation in the brain aging and neurodegenerative diseases: A role of senescent cells. Neural Regen. Res..

[44] Sikora E., Bielak-Zmijewska A., Dudkowska M., Krzystyniak A., Mosieniak G., Wesierska M., Wlodarczyk J. (2021). Cellular Senescence in Brain Aging. Front. Aging Neurosci..

[45] Schmidt R., Verstraete E., de Reus M.A., Veldink J.H., van den Berg L.H., van den Heuvel M.P. (2014). Correlation between structural and functional connectivity impairment in amyotrophic lateral sclerosis. Hum. Brain Mapp..

[46] Geloso M.C., Corvino V., Marchese E., Serrano A., Michetti F., D’Ambrosi N. (2017). The Dual Role of Microglia in ALS: Mechanisms and Therapeutic Approaches. Front. Aging Neurosci..

[47] Thonhoff J.R., Simpson E.P., Appel S.H. (2018). Neuroinflammatory mechanisms in amyotrophic lateral sclerosis pathogenesis. Curr. Opin. Neurol..

[48] Spiller K.J., Restrepo C.R., Khan T., Dominique M.A., Fang T.C., Canter R.G., Roberts C.J., Miller K.R., Ransohoff R.M., Trojanowski J.Q. (2018). Microglia-mediated recovery from ALS-relevant motor neuron degeneration in a mouse model of TDP-43 proteinopathy. Nat. Neurosci..

[49] Bendat J.S., Piersol A.G. (1986). Random Data.

[50] Welch P. (1967). The use of fast Fourier transform for the estimation of power spectra: A method based on time averaging over short, modified periodograms. IEEE Trans. Audio Electroacoust..

[51] https://web.archive.org/web/20070831220654/http://www.codegear.com/products/cppbuilder.

