# Supplementary material for: Age-Related Modifications of Electroencephalogram Coherence in Mice Models of Alzheimer’s Disease and Amyotrophic Lateral Sclerosis
Source: Biomedicines. 2023 Apr 11;11(4):1151. doi: 10.3390/biomedicines11041151 (PMC10136324; doi:10.3390/biomedicines11041151)
Supplement: Supplementary file 1 [file biomedicines-11-01151-s001.zip › Figure S1.pdf]

Figure S1

Short description of special software developed in this study for calculation of EEG coherence.

The coherence $C_{xy}(f)$ between two signals *x*(*t*) and *y*(*t*) was calculated as:

$$C_{xy}(f) = \frac{\left|G_{xy}(f)\right|^2}{G_{xx}(f) * G_{yy}(f)},$$

where $G_{xy}(f)$ is the cross-power spectrum between *x(t)* and *y(t)*, $G_{xx}(f)$ and $G_{yy}(f)$ represent the auto-power spectrum of *x*(*t*) and *y*(*t*), respectively, *f* is a frequency and *t* is a time [1]. The spectra were calculated as:

$$G_{xy}(f) = X(f) * Y^*(f),\ G_{xx}(f) = X(f) * X^*(f),\ G_{yy}(f) = Y(f) * Y^*(f),$$

where $X(f), Y(f)$ are Fourier transforms of *x*(*t*) and *y(t)* signals, $X^*(f), Y^*(f)$ are complex conjugates of $X(f), Y(f)$.

Thereafter, the Welch's averaging technique was applied [2]. The signal was divided into *N* equal length segments overlapped by one half, and then spectra $X_k(f), Y_k(f)$ were calculated for each of k-segments. After that the averaged coherence function was estimated as:

$$C_{xy}(f) = \frac{\left|\sum_k X_k(f) * Y_k^*(f)\right|^2}{\left(\sum_k \left|X_k(f)\right|^2\right)\left(\sum_k \left|Y_k(f)\right|^2\right)}$$

For coherence calculation, we developed special software based on Embarcadero C++ Builder development environment [3].

References:

1. Bendat, J.S.; Piersol, A.G. Random Data, Wiley-Interscience, 1986
2. Welch, P. The use of fast Fourier transform for the estimation of power spectra: a method based on time averaging over short, modified periodograms. *IEEE Trans. Audio Electroacoust.* **1967**, 15, 70–73. doi: 10.1109/TAU.1967.1161901
3. https://web.archive.org/web/20070831220654/http://www.codegear.com/products/cppbuilder
