# Supplementary material for: Age-Related Modifications of Electroencephalogram Coherence in Mice Models of Alzheimer’s Disease and Amyotrophic Lateral Sclerosis
Source: Biomedicines. 2023 Apr 11;11(4):1151. doi: 10.3390/biomedicines11041151 (PMC10136324; doi:10.3390/biomedicines11041151)
Supplement: Supplementary file 1 [file biomedicines-11-01151-s001.zip › Figure S2.pdf]

Figure S2

Two-way ANOVA analysis of differences between EEG coherence distributions in 5xFAD- and WT_{5xFAD}- mice of different ages.

	**5xFAD- vs. WT_{5xFAD}- mice**							
**Age, months**	**6**		**9**		**12**		**18**	
**Areas/Coherence**	F_{138}	*p*	F_{90}	*p*	F_{84}	*p*	F_{72}	*p*
MCsin-HPCsin	17.9	<0.001	7.8	0.006	11.2	0.001	1.1	0.299
MCsin-VTAsin	105	<0.001	233	<0.001	11.1	0.001	0.3	0.588
MCsin-SNdex	107	<0.001	90.7	<0.001	10.8	0.002	3.6	0.063
HPCsin-VTAsin	163	<0.001	29.2	<0.001	11.5	0.001	13.4	<0.001
HPCsin-SNdex	96.4	<0.001	36.6	<0.001	10.9	0.001	0.6	0.440
VTAsin-SNdex	46.3	<0.001	115	<0.001	17.7	<0.001	9.4	0.003
