# Supplementary material for: Age-Related Modifications of Electroencephalogram Coherence in Mice Models of Alzheimer’s Disease and Amyotrophic Lateral Sclerosis
Source: Biomedicines. 2023 Apr 11;11(4):1151. doi: 10.3390/biomedicines11041151 (PMC10136324; doi:10.3390/biomedicines11041151)
Supplement: Supplementary file 1 [file biomedicines-11-01151-s001.zip › Figure S3.pdf]

Figure S3

Two-way ANOVA analysis of differences between EEG coherence distributions in FUS- and WT_{FUS}-mice of different ages

	FUS- vs. WT_{FUS}- mice			
Age, months	**2**		**5**	
Areas/Coherence	F_{72}	***p***	F_{66}	***p***
MCsin-Ptsin	*7.3*	*0.008*	*5.1*	*0.028*
MCsin-MSdex	*16.3*	*<0.000*	*2.0*	*0.161*
MCsin-Ptdex	*21.8*	*<0.000*	*3.8*	*0.056*
Ptsin-MCdex	*15.1*	*<0.000*	*0.8*	*0.362*
Ptsin-Ptdex	*12.7*	*<0.000*	*3.0*	*0.086*
MCdex-Ptdex	*23.2*	*<0.000*	*0.9*	*0.353*
